# Regioselective Synthesis of New 2-(*E*)-Cyano(thiazolidin-2-ylidene)thiazoles

**DOI:** 10.3390/molecules14114849

**Published:** 2009-11-25

**Authors:** Mehdi Bakavoli, Hamid Beyzaie, Mohammad Rahimizadeh, Hossein Eshghi, Reza Takjoo

**Affiliations:** 1 Department of Chemistry, School of Sciences, Ferdowsi University of Mashhad, 91775-1436 Mashhad, Iran; E-Mails: hamidchem@yahoo.com (H.B.); rahimizh@yahoo.com (M.R.); heshghi@ferdowsi.um.ac.ir (H.E.); 2 Department of Chemistry, School of Science, Islamic Azad University, Mashhad Branch, Mashhad, Iran; E-Mail: rezatakjoo@yahoo.com

**Keywords:** thiazole, thiazolidine, Hantzsch’s synthesis, heterocyclization, regioselective synthesis, X-ray crystallography

## Abstract

Cyclocondensation of 2-[bis(methylthio)methylene]malononitrile (**1**) and cysteamine (**2**) afforded 2-(thiazolidin-2-ylidene)malononitrile (**3**). This compound on treatment with NaSH gave the corresponding thioamide derivative **4a** in a regioselective manner on the basis of its single crystal X-ray diffraction analysis. Reaction of this compound with several α-bromocarbonyl compounds gave new 2-(*E*)-cyano(thiazolidin-2-ylidene)thiazoles **5a-g**.

## Introduction 

Thiazole is an important scaffold in heterocyclic chemistry and 1,3-thiazole ring is present in many pharmacologically active substances [1]. For example thiazole-5-ylacetic acid derivatives possess strong anti-inflammatory activity [2]. Other compounds containing the thiazole ring have been reported as being histamine H3 antagonists [3], with herbicidal [4], antitumoral [5] and selective cardiodepressant activities [6].

Several methods for the synthesis of thiazole derivatives have been developed [7,8,9,10,11], the most widely used method being the Hantzsch’s synthesis utilizing thioamides and α-halocarbonyl compounds as the starting materials [12]. The preparation of thioamides has been reviewed elsewhere [13]. One procedure involves nitriles which can be converted to thioamides through treatment with elemental sulfur, S_8_ [14,15], gaseous hydrogen sulfide in the presence of anion exchange resin (Dowex 1X8, SH- from) at room temperature [16], sodium hydrosulfide hydrate and magnesium chloride hexahydrate in DMF or methanol [17], ammonium sulfide in methanol [18] and phosphorus pentasulfide [19].

Our interest in the synthesis of heterocyclic compounds of biologically importance has encouraged us to study the synthesis of some new functionalized thiazoles. In this context we wish to report on the reactions and single crystal X-ray diffraction analysis of a newly synthesized thioamide which on treatment with various α-bromocarbonyl compounds afforded some new 2-(*E*)-cyano(thiazolidin-2-ylidene)thiazoles.

## Results and Discussion 

**Scheme 1:**
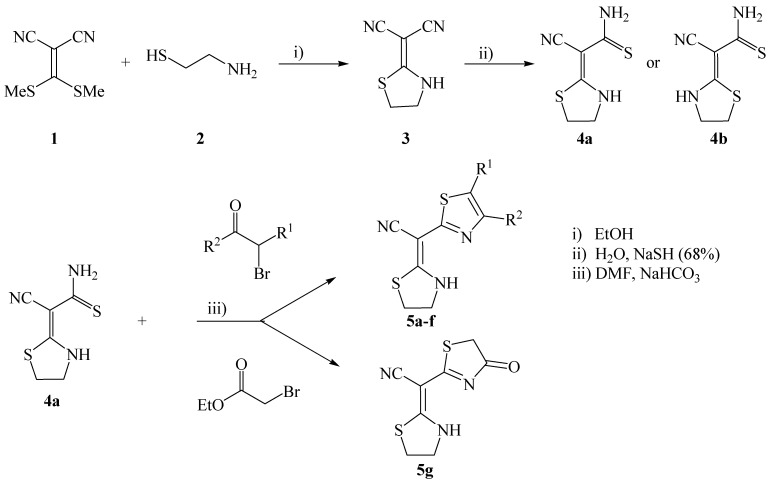
Synthesis of 2-(*E*)-cyano(thiazolidin-2-ylidene)thiazoles **5a-g**. For substituents R^1^ and R^2^ see Table 1.

2-(*E*)-Cyano(thiazolidin-2-ylidene)thiazoles **5a-g** were prepared in a three-step procedure starting from the dinitrile **3** (Scheme 1). Cyclocondensation of 2-[bis(methylthio)methylene]malononitrile (**1**) and cysteamine (**2**) in ethanol afforded thiazolidine **3**, which was filtered off and thionated by sodium hydrosulfide hydrate in water to give 2-cyano-2-(thiazolidin-2-ylidene)ethanethioamide (**4**) that can form either *E* and/or *Z* geometric isomers **4a,b**. 

**Figure 1:**
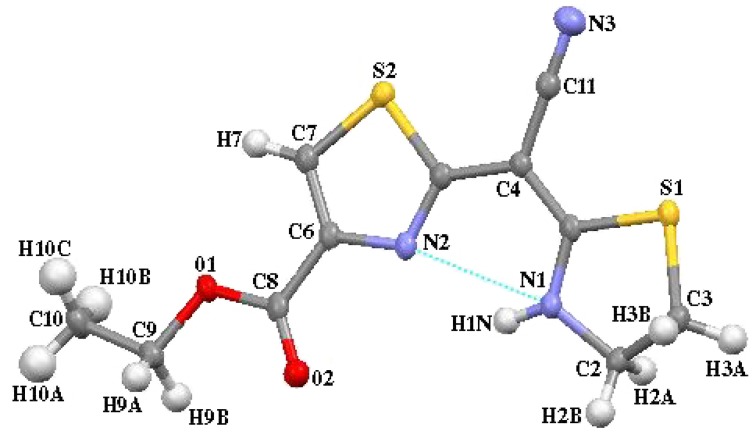
Molecular plot of **5a** with the thermal ellipsoids representing 50% probability.

**Figure 2:**
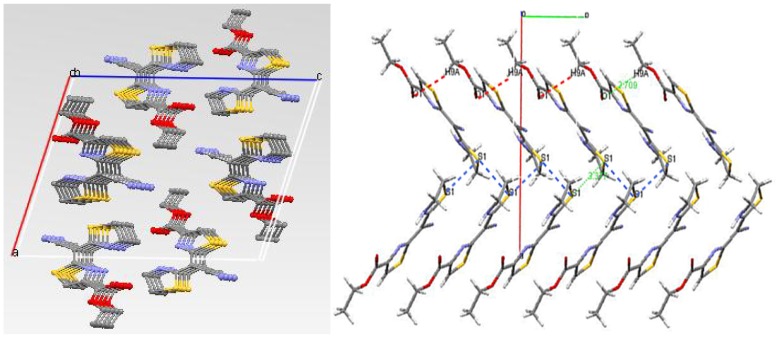
Molecular packing of **5a** showing Van der Waals as dashed lines along b axis.

For exact determination of these geometric isomers **4a,b**, single crystal X-ray analysis would seem to be preferable to other available techniques but all our efforts to prepare a suitable single crystal from compound **4** were unsuccessful, but instead we obtained a single crystal from the derivative ethyl 2-(*E*)-cyano(thiazolidin-2-ylidene)methyl)thiazole-4-carboxylate (**5a**). X-ray quality crystals were grown from ethanol with slow evaporation. A crystal structure was determined for product **5a** and is shown in Figure 1. The molecule exists in the *E* geometric form with respect to C1-C4 bond. This observation clearly indicates that compound **4** must have the *E* isomeric structure **4a**. The packing diagram of **5a** shows that there is intermolecular hydrogen bonding between the hydrogen atom on the N1 and the N2 atom. Another intramolecular interaction is Van der Waals interaction between molecules. The high contact interaction between S1…S1 (3.777 Å) and O1…H9A (2.709 Å) gives rise to crystal growth expansion along b axis (see Figure 2). 

The bond lengths of five-membered ring C5S2C7C6N2 are shorter than that ring C1S1C3C2N1, confirming the aromacity of the former ring. In this compound the C4C11N3 bond and the aromatic ring are in the same plane.

The thioamide **4a** was treated with α-bromocarbonyl compounds in DMF to afford the new 2-(*E*)-cyano(thiazolidin-2-ylidene)thiazoles **5a-g** (Table 1). The structures of compounds **3**, **4a** and **5a-g** were deduced from their elemental analyses and ^1^H-NMR spectral data. The ^1^H-NMR spectra of compounds **3**, **4a** and **5a-g** showed triplet signals at 3.47-3.63 ppm and 3.99-4.32 ppm (*J* ~ 7.6 Hz) due to the two methylene groups of the thiazolidine rings, and broad signals due to the NH group at 6.84-12.17 ppm. The ^13^C-NMR spectrum of **4a** showed signals due to nitile and thiocarbonyl groups at 119.42 ppm and 189.56 ppm. IR spectroscopy also confirmed the structure of products. 

## Experimental 

### General

All of the melting points are uncorrected and were recorded on an Electrothermal type 9100 melting point apparatus. The mass spectra observed on a Varian Mat CH-7 at 70 ev. The infrared spectra were determined on 4300 Shimadzu spectrometer and only noteworthy absorptions are listed. The ^1^H-NMR spectra were recorded on a Bruker AC 100 spectrometer. The ^13^C-NMR spectrum was recorded on a Bruker Avance DRX-500 Fourier transform spectrometer. Chemical shifts are reported in ppm downfield from TMS as internal standard. All solvents and chemicals were of research grade and were used as obtain from Merck. Elemental analyses were performed on a Thermo Finnigan Flash EA microanalyzer. Compound **1** was prepared according to the procedure reported in the literature [20].

### Synthesis of 2-(thiazolidin-2-ylidene)malononitrile *(**3**)*

A suspension of 2-[bis(methylthio)methylene]malononitrile **1** (3.4 g, 0.02 mol) and cysteamine **2** (1.54 g, 0.02 mol) in ethanol (96%) (40 mL) was stirred at room temperature for 4 h. The white powder of **3** that precipitated was collected by suction filtration, washed with ethanol (2 × 10 mL), and dried in air to give 2.2 g (73%) of **3**, mp 209-212 °C; IR (KBr): 3,195, 2,207, 1,571 cm^-1^; ^1^H-NMR (CDCl_3_) δ 3.48 (t, *J* = 6.9 Hz, 2H, SCH_2_), 3.99 (t, *J* = 6.9 Hz, 2H, NCH_2_), 6.84 (s, 1H, NH); MS (70 eV) m/z: 151 (M+); Anal. Calcd. for C_6_H_5_N_3_S: C, 47.66; H, 3.34; N, 27.79; S, 21.21. Found: C, 47.56; H, 3.43; N, 27.71; S, 21.30.

### Synthesis of (E)-2-cyano-2-(thiazolidin-2-ylidene)ethanethioamide *(**4a**)*

A solution of thiazolidine **3** (1.5 g, 0.01 mol) and sodium hydrosulfide hydrate (68%) (1.65 g, 0.02 mol) in water (10 mL) was heated at 50 °C for 22 h. The reaction mixture was cooled to room temperature and the product was precipitated by addition of acetic acid. The precipitate was collected by suction filtration, washed with ethanol (2 × 5 mL), dried in air, and recrystallized from CH_3_CN, to give 1.21g (65%) of **4a** as white needles, mp 217-219 °C; IR (KBr): 3,435, 3,320, 2,182, 1,613, 1,570 cm^-1^; ^1^H-NMR (acetone-*d_6_*) δ: 3.61 (t, *J* = 7.1 Hz, 2H, SCH_2_), 4.32 (t, *J* = 7.1 Hz, 2H, NCH_2_), 7.50 (s, 2H, NH_2_), 12.17 (1H, s, NH); ^13^C-NMR (DMSO*-d_6_*) δ: 30.28, 52.86, 75.45, 119.42, 175.53, 189.56; MS (70 eV) m/z: 185 (M^+^); Anal. Calcd. for C_6_H_7_N_3_S_2_: C, 38.90; H, 3.81; N, 22.68; S, 34.61. Found: C, 38.95; H, 3.74; N, 22.76; S, 34.55.

### General procedure for the synthesis of 2- (E)-cyano(thiazolidin-2-ylidene)thiazoles ***5a-g***

A suspension of thioamide **4a** (1.85 g, 1 mmol), the appropriate α-bromocarbonyl (1 mmol) and sodium bicarbonate (0.84 g, 1 mmol) in DMF (1 mL) was stirred at room temperature for 2-8 h. After dilution with water (5 mL), the solid obtained was filtered off, washed with water (1 × 5 mL) and ethanol (1 × 5 mL), dried in air, and recrystallized from CH_3_CN, to give **5a-g** in 71–82% yield.

*Ethyl 2-[(E)-cyano(thiazolidin-2-ylidene)methyl]thiazole-4-carboxylate* (**5a**): mp 264-267 °C; IR (KBr): 3,430, 2,186, 1,597, 1,561 cm^-1^; ^1^H-NMR (DMSO-*d_6_*) δ: 1.34 (t, *J* = 6.6 Hz, 3H, CH_3_), 3.52 (t, *J* = 7.2 Hz, 2H, SCH_2_), 4.12 (t, *J* = 7.2 Hz, 2H, NCH_2_), 4.31 (q, *J* = 6.6 Hz, 2H, OCH_2_), 8.24 (s, 1H, C=C-H), 9.93 (s, 1H, NH); MS (70 eV) m/z: 281 (M^+^); Anal. Calcd. for C_11_H_11_N_3_O_2_S_2_: C, 46.96; H, 3.94; N, 14.94; S, 22.79. Found: C, 47.07; H, 4.02; N, 14.90; S, 22.63.

*(E)-2-(4-Methylthiazol-2-yl)-2-(thiazolidin-2-ylidene)acetonitrile* (**5b**): mp 166-168 °C; IR (KBr): 3,438, 2,180, 1,563 cm^-1^; ^1^H-NMR (DMSO-*d_6_*) δ: 2.32 (s, 3H, CH_3_), 3.47 (t, *J* = 7.7 Hz, 2H, SCH_2_), 4.01 (t, *J* = 7.7 Hz, 2H, NCH_2_), 6.91 (s, 1H, C=C-H), 9.94 (s, 1H, NH); MS (70 eV) m/z: 223 (M^+^); Anal. Calcd. for C_9_H_9_N_3_S_2_: C, 48.41; H, 4.06; N, 18.82; S, 28.71. Found: C, 48.40; H, 4.10; N, 18.74; S, 28.76.

*(E)-2-(4-Phenylthiazol-2-yl)-2-(thiazolidin-2-ylidene)acetonitrile* (**5c**): mp 181-184 °C; IR (KBr): 3,438, 2,187, 1,563 cm^-1^; ^1^H-NMR (DMSO-*d_6_*) δ: 3.53 (t, *J* = 7.9 Hz, 2H, SCH_2_), 4.12 (t, *J* = 7.9 Hz, 2H, NCH_2_), 7.41, 8.05 (5H, Ph), 7.85 (s, 1H, C=C-H), 9.81 (s, 1H, NH); MS (70 eV) m/z: 285 (M^+^); Anal. Calcd. for C_14_H_11_N_3_S_2_: C, 58.92; H, 3.88; N, 14.73; S, 22.47. Found: C, 58.94; H, 3.77; N, 14.41; S, 22.78.

*(E)-2-(4-(4-Nitrophenyl)thiazol-2-yl)-2-(thiazolidin-2-ylidene)acetonitrile* (**5d**): mp 264-267 °C; IR (KBr): 3,433, 2,193, 1,569 cm^-1^; ^1^H NMR (DMSO-*d_6_*) δ: 3.62 (t, *J* = 7.8 Hz, 2H, SCH_2_), 4.13 (t, *J* = 7.8 Hz, 2H, NCH_2_), 8.15 (s, 1H, C=C-H), 8.31 (s, 4H, Ph), 9.65 (s, 1H, NH); MS (70 eV) m/z: 330 (M^+^); Anal. Calcd. for C_14_H_10_N_4_O_2_S_2_: C, 50.90; H, 3.05; N, 16.96; S, 19.41. Found: C, 51.12; H, 2.97; N, 17.02; S, 19.56.

*(E)-2-(5-Acetyl-4-methylthiazol-2-yl)-2-(thiazolidin-2-ylidene)acetonitrile* (**5e**): mp 255-257 °C; IR (KBr): 3,421, 2,191, 1,648, 1,557 cm^-1^; ^1^H-NMR (DMSO-*d_6_*) δ: 2.52 (s, 3H, COCH_3_), 2.65 (s, 3H, CH_3_), 3.52 (t, *J* = 7.7 Hz, 2H, SCH_2_), 4.12 (t, *J* = 7.7 Hz, 2H, NCH_2_), 10.13 (s, 1H, NH); MS (70 eV) m/z: 265 (M^+^); Anal. Calcd. for C_11_H_11_N_3_OS_2_: C, 49.79; H, 4.18; N, 15.84; S, 24.16. Found: C, 49.68; H, 4.02; N, 15.60; S, 24.42.

*Ethyl 2-((E)-cyano(thiazolidin-2-ylidene)methyl)-4-methylthiazole-5 carboxylate* (**5f**): mp 253-255 °C; IR (KBr): 3,443, 2,190, 1,706, 1,562 cm^-1^; ^1^H-NMR (DMSO-*d_6_*) δ: 1.26 (t, *J* = 7.1 Hz, 3H, CH_2_CH_3_), 2.60 (s, 3H, CH_3_), 3.57 (t, *J* = 7.6 Hz, 2H, SCH_2_), 4.06 (t, *J* = 7.6 Hz, 2H, NCH_2_), 4.16 (q, *J* = 7.1 Hz, 2H, OCH_2_), 10.13 (s, 1H, NH); MS (70 eV) m/z: 295 (M^+^); Anal. Calcd. for C_12_H_13_N_3_O_2_S_2_: C, 48.80; H, 4.44; N, 14.23; S, 21.71. Found: C, 48.76; H, 4.27; N, 14.05; S, 21.77.

*(2E)-2-(4,5-Dihydro-4-oxothiazol-2-yl)-2-(thiazolidin-2-ylidene)acetonitrile* (**5g**): mp 248-252 °C; IR (KBr): 3,422, 2,193, 1,688, 1,573 cm^-1^; ^1^H-NMR (DMSO-*d_6_*) δ: 3.63 (t, *J* = 7.8 Hz, 2H, SCH_2_), 4.01 (s, 2H, COCH_2_), 4.12 (t, *J* = 7.8 Hz, 2H, NCH_2_), 10.53 (s, 1H, NH); MS (70 eV) m/z: 225 (M^+^); Anal. Calcd. for C_8_H_7_N_3_OS_2_: C, 42.65; H, 3.13; N, 18.65; S, 28.46. Found: C, 42.74; H, 3.00; N, 18.46; S, 28.68.

### Crystal data for ***5a***

Crystallographic data and details of **5a** are presented in Table 2 and Table 3. The X-ray diffraction data for crystals of **5a** were collected on Bruker AXS Smart Apex II CCD diffractometer at 100 K. The raw intensity data frames were integrated with the SAINT program, which also applied corrections [21]. Data were corrected for absorption effects by the SADABS procedure [22]. The software package SHELXTL v6.12 was used for the space group determination, structure solution and refinement. The structures were solved by direct methods (SHELXS-97) [23], completed with difference Fourier syntheses and refined with full-matrix least-squares using SHELXL-97 minimizing w =1/[σ^2^(F_o_^2^)+(0.0440P)^2^+0.8400P] where P=(F_o_^2^+2F_c_^2^)/3. CCDC 711896 contains the supplementary crystallographic data for this article. These data can be obtained free of charge from the Cambridge Crystallographic Data Center via www.ccdc.cam.ac.uk/conts/retrieving.html. 

## Conclusions 

We have reported synthesis of novel 2-(*E*)-cyano(thiazolidin-2ylidine)thiazoles from dinitrile **3** as a starting material. Application of these compounds to the synthesis of other heterocyclic ring systems is currently under investigation and the results will be published in due course.

## Figures and Tables

**Table 1:** Results of reaction **4a** and α-bromocarbonyl compounds in DMF at room temperature.

Entry	R^1^	R^2^	Substrate	α-Bromocarbonyl	Time, h	Yield, %
1	H	CO_2_Et	**5a**	Ethyl bromopyruvate	2	71
2	H	CH_3_	**5b**	Bromoacetone	2	73
3	H	C_6_H_5_	**5c**	Phenacyl bromide	2	79
4	H	*p*-NO_2_C_6_H_4_	**5d**	*p*-Nitrobromoacetophenone	2	82
5	COCH_3_	CH_3_	**5e**	3-Bromo acetylacetone	2	77
6	CO_2_Et	CH_3_	**5f**	Ethyl 2-bromoacetoacetate	2	77
7	-	-	**5g**	Ethyl bromoacetate	8	75

**Table 2:** Crystallographic data for compound **5a**.

Empirical formula	C_11_H_11_N_3_O_2_S_2_
Formula weight	281.35
Temperature	100(2) K
Crystal system	Monoclinic
Crystal size	0.30 × 0.08 × 0.04 mm^3^
Space group	*P*2_1_/*n*
*a*	14.9127(16) Å
*b* *c*	4.6129(5) Å 18.658(2) Å
*β*	106.175(2)°
*V*	1232.7(2) Å^3^
*Z*	4
*D_calc_*	1.516 Mgm^-3^
*µ*	0.429 mm-1
*F*(000)	584
*θ* range	1.55 to 30.66°
Index ranges	-21<=h<=21, -6<=k<=6, -26<=l<=26
Reflections collected	15302
Data/restraints/parameters	3802/0/164
Goodness-of-fit on F2	1.006
Radiation	Mo K_α_ 0.71073 (*λ*, Å)
Independent reflections	3802 [R(int) = 0.0473]
Completeness to theta = 30.66°	99.2 %
Absorption correction	Semi-empirical from equivalents
Refinement method	Full-matrix least-squares on F^2^
Final R indices [for 4865 rfln. with *I>2σ(I)*]	R1 = 0.0419, wR2 = 0.0947
R indices (all data)	R1 = 0.0588, wR2 = 0.1022
Largest diff. peak and hole	0.368 and -0.257 e.Å^-3^

**Table 3:** Selected bond lengths [Å] and angles [°] for compound **5a**.

S(1)-C(1)	1.7507(17)	C(1)-S(1)-C(3)	90.58(8)
S(1)-C(3)	1.8222(19)	C(7)-S(2)-C(5)	89.28(8)
S(2)-C(7)	1.7141(18)	C(8)-O(2)-C(9)	115.60(14)
S(2)-C(5)	1.7459(17)	C(1)-N(1)-C(2)	115.26(14)
O(1)-C(8)	1.207(2)	C(5)-N(2)-C(6)	110.17(14)
O(2)-C(8)	1.342(2)	N(1)-C(1)-C(4)	125.56(15)
O(2)-C(9)	1.462(2)	N(1)-C(1)-S(1)	112.39(12)
N(1)-C(1)	1.333(2)	C(4)-C(1)-S(1)	122.04(13)
N(1)-C(2)	1.463(2)	N(1)-C(2)-C(3)	104.77(14)
N(2)-C(5)	1.315(2)	C(2)-C(3)-S(1)	104.89(12)
N(2)-C(6)	1.382(2)	C(1)-C(4)-C(11)	119.36(15)
N(3)-C(11)	1.153(2)	C(1)-C(4)-C(5)	121.88(15)
C(1)-C(4)	1.385(2)	C(11)-C(4)-C(5)	118.75(15)
C(2)-C(3)	1.522(3)	N(2)-C(5)-C(4)	125.14(15)
C(4)-C(11)	1.418(2)	N(2)-C(5)-S(2)	114.32(12)
C(4)-C(5)	1.443(2)	C(4)-C(5)-S(2)	120.53(13)
C(6)-C(7)	1.358(2)	C(7)-C(6)-N(2)	116.01(16)
C(6)-C(8)	1.484(2)	C(7)-C(6)-C(8)	125.49(16)
C(9)-C(10)	1.506(3)	N(2)-C(6)-C(8)	118.41(15)
		C(6)-C(7)-S(2)	110.22(13)
		O(1)-C(8)-O(2)	124.65(16)
		O(1)-C(8)-C(6)	124.04(16)
		O(2)-C(8)-C(6)	111.30(15)
		O(2)-C(9)-C(10)	107.40(15)
		N(3)-C(11)-C(4)	179.5(2)
